# A novel causative functional mutation in *GATA6* gene is responsible for familial dilated cardiomyopathy as supported by in silico functional analysis

**DOI:** 10.1038/s41598-022-13993-6

**Published:** 2022-08-12

**Authors:** Afrouz Khazamipour, Nazanin Gholampour-Faroji, Tina Zeraati, Farveh Vakilian, Aliakbar Haddad-Mashadrizeh, Majid Ghayour Mobarhan, Alireza Pasdar

**Affiliations:** 1 Department of Medical Genetics and Molecular Medicine, Faculty of Medicine, Mashhad University of Medical Sciences, Mashhad, Iran; 2 Biotechnology Department, Iranian Research Organization for Science and Technology (IROST), Tehran, Iran; 3 Medical Genetics Research Centre, Faculty of Medicine, Mashhad University of Medical Sciences, Mashhad, Iran; 4 Atherosclerosis Prevention Research Centre, Faculty of Medicine, Mashhad University of Medical Sciences, Mashhad, Iran; 5 Industrial Biotechnology Research Group, Institute of Biotechnology, Ferdowsi University of Mashhad, Mashhad, Iran; 6 Metabolic Syndrome Research Centre, Faculty of Medicine, Mashhad University of Medical Sciences, Mashhad, Iran; 7 Division of Applied Medicine, Medical School, University of Aberdeen, Foresterhill, Aberdeen, UK; 8 Bioinformatics Research Centre, Mashhad University of Medical Sciences, Mashhad, Iran

**Keywords:** Biotechnology, Genetics

## Abstract

Dilated cardiomyopathy (DCM), one of the most common types of cardiomyopathies has a heterogeneous nature and can be seen in Mendelian forms. Next Generation Sequencing is a powerful tool for identifying novel variants in monogenic disorders. We used whole-exome sequencing (WES) and Sanger sequencing techniques to identify the causative mutation of DCM in an Iranian pedigree. We found a novel variant in the *GATA6* gene, leading to substituting Histidine by Tyrosine at position 329, observed in all affected family members in the pedigree, whereas it was not established in any of the unaffected ones. We hypothesized that the H329Y mutation may be causative for the familial pattern of DCM in this family. The predicted models of *GATA6* and H329Y showed the high quality according to PROCHECK and ERRAT. Nonetheless, simulation results revealed that the protein stability decreased after mutation, while the flexibility may have been increased. Hence, the mutation led to the increased compactness of *GATA6*. Overall, these data indicated that the mutation could affect the protein structure, which may be related to the functional impairment of *GATA6* upon H329Y mutation, likewise their involvement in pathologies. Further functional investigations would help elucidating the exact mechanism.

## Introduction

Dilated cardiomyopathy (DCM) is a primary disorder of the cardiovascular system. DCM is characterized by left cardiac enlargement owing to left ventricular dilation as well as reduced systolic function, which is not secondary to ischemia, valvular disease, and hypertension. It may result in heart failure with a noticeable rate of morbidity and mortality^1–3^. The impaired systolic function is observed in the initial stages; however, diastolic dysfunction in association with reduced ejection fraction will be present in the advance stages^4^. DCM has a prevalence of one case out of 250 individuals^5^. It is associated with an increased morbidity and mortality rate, as it may result in heart failure leading to sudden cardiac death (SCD)^6,7^ with a reported frequency of 46%^7^. DCM results from a broad spectrum of aetiologies, about 50% of which are remained idiopathic. According to the literature, there are more than 60 genes capable of being involved in the pathogenesis of DCM, where a positive genetic background is considered in most patients^6^. Inheritance of DCM is complex with incomplete penetrance, high variability in disease onset and progression, and genetic heterogeneity^3,7,8^. Genetic evaluation seems to be beneficial for the patients not only to predict the prognosis and set the management strategy, but also to provide a family screening and make prediction on the risk of recurrence amongst the next generation^6^. Modern molecular genetic techniques have become popular amongst genetic specialists, for their capability of identifying various genes associating with numerous diseases^9–11^. The new generation of sequencing method called "Next-generation sequencing" (NGS) is known for its cost-effective, high-power, and high-speed function in providing the genetic evaluation of a growing number of disorders; It has enhanced our knowledge about the impact of different genes and variants in the case of disease formation and progression^12^. In addition, whole-exome sequencing (WES) is globally used as a standard diagnostic test, achieving the molecular diagnosis of rare Mendelian disorders with a high accuracy and sensitivity. Furthermore, its beneficial role in evaluating the members of families, many of whom are affected by a specific genetic disease, have been approved through research studies^6,10^.

The human *GATA6* gene is mapped to chromosome 18q11.1 to q11.2, and it consists of six introns and seven exons placed apart from one another. In humans, *GATA6* gene expression in the heart, ovary, pancreas, liver, lung, adrenal, central nervous system, and vascular smooth muscle cells (VSMCs) has been confirmed, however, its expression in the heart and lung of human foetus is significantly higher^13,14^. The expression of the *GATA6* gene in the heart during embryogenesis has been approved in the literature, and the gene has been detected in different human primary endothelial cells (ECs), VSMCs, and vascular ECs in mice. The only member of the *GATA* family expressed in VSMCs is *GATA6*, and it is also involved in the maintenance of the differentiation phenotype of VSMCs^14–16^.

The role of *GATA6* variants in the pathogenesis of cardiac arrhythmia such as atrial fibrillation and congenital cardiovascular malformations have been established^17–21^. The definite number of mutations in this gene, which may be responsible for DCM development is not recorded in the literature although, to date, there are over 30 known *GATA6* mutations reported in various forms of CHD. However, the exact mechanisms by which mutations in *GATA6* and other gene family members such as *GATA4* and *GATA5* lead to CHD are still unclear^22^.

The *GATA* family of transcription factors is a highly conserved DNA binding domain, in which there are two zinc finger structures. The structures prefer binding to a 5′-(A/T)*GATA*(A/G)-3′ motif within the regulatory region of target genes. The *GATA* family consists of six components (*GATA1*-*GATA6*) in vertebrates, all of which are mainly involved in growth regulation, differentiation, and the survival of different cells as well as the maintenance of the homeostasis. Besides, the components are expressed in several cells and tissues, such as hematopoietic cells.

In this study, we used WES and Sanger sequencing to investigate the genetic background of a family consisted of members showing a familial pattern of FDCM (familial dilated cardiomyopathy) manifested with early-onset heart disease. Recently, considerable computational efforts have been implicated in appraising the functional and structural outcomes resulted from novel sequence variations. A growing number of in silico tools appeared beneficial, identifying deleterious mutations related to diseases^23,24^. The state-of-the-art molecular dynamics (MD) simulation has recently been showed to be accountable in detecting the significant disease-causing mutations and the structural consequences of the mutation which has been studied by many researchers using molecular dynamics simulations analysis^25–27^. There have been several essays, reporting the probable explanations of the changes in the wild-type and mutant state dynamics, in addition to their association with protein-function at the atomic level^28–30^. To provide evidence for possible effects of the candidate variant, molecular dynamics simulation approach was also performed in order to clearly represent the structural variations with time, beside evaluating the varied structural characteristics related to interacting behaviours of the wild-type of *GATA6* and mutant.

## Materials and methods

### Study population

**Figure 1:**
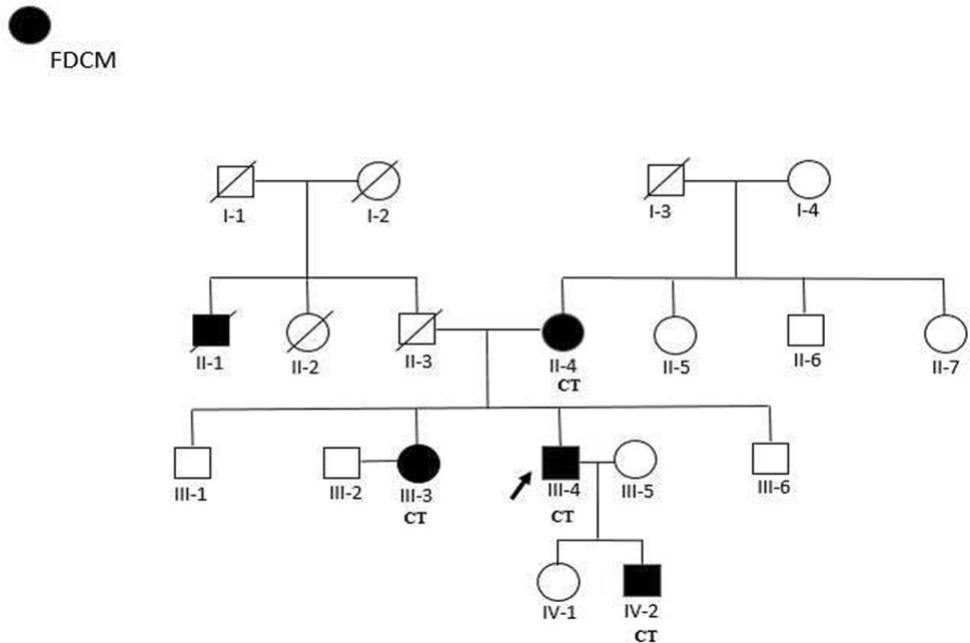
The Pedigree of a family suffering early-onset FDCM. WES was performed for III-4 (proband); a novel heterozygous variant (c.985C > T, p.H329Y) in *GATA6* was identified in the III-4, IV-2, III-3, and II-4 patients. Healthy individuals (non-affected such as III-1 and III-5) had normal genotype (CC). Deceased members are shown by slashes. Solid symbols are indicating the family members diagnosed with FDCM. Genotypes are shown as: Wile-type allele = C_,_ Mutant allele = T.

The study was approved by the Ethics Committee of Mashhad University of Medical Sciences (ethical code IR.MUMS.MEDICAL.REC.1399.218). All the methods and protocols were performed in accordance with the guidelines and regulations of Mashhad University of Medical Sciences and the Declaration of Helsinki Ethical Principles for Medical Research involving human subjects. After obtaining informed consent, we recruited the members of a family, most of whom were diagnosed with FDCM based on the findings of cardiac MRI and echocardiography, and the cardiologist`s clinical judgment. They presented to our clinic in Mashhad with an unusual form of early-onset cardiomyopathy in adolescence. The pedigree was suggesting of an autosomal dominant pattern of inheritance amongst family members (Fig. 1). After recording their clinical history, laboratory data (Table 1), and imaging findings, blood samples were taken from each participant (5 ml venous blood in collecting-tubes containing EDTA anticoagulants) in order to extract the genomic DNA. Moreover, 50 healthy persons with no cardiac symptoms were selected from the general population in the same area to compare the allele frequencies of the candidate variant.

**Table 1:** The clinical, echocardiography and biochemical characteristics of the both unaffected and affected members.

No ID	Sex	Age of onset DCM	Symptoms	NYHA class	LVEF (%)	LVEDD (cm)	LVESD (cm)	IVSD (cm)	pro-BNP (pg/mL)	HTN	Smoking
II-4	F	20	Dyspnea, fatigue, orthopnea	II	35	5.9	4	0.7	451.5	Yes	No
III-3	F	18	Dyspnea, fatigue	III	20	6.4	5.6	0.6	426	No	No
III-4	M	17	Dyspnea, fatigue, orthopnea	III	25	6.1	6.1	0.9	311	No	No
IV-2	M	16	Dyspnea, fatigue, chest pain	II	30	5.8	4.5	0.9	122.5	No	No
III-1	F	No-affected	None	None	55	4.2	3.2	1	100	No	No
III-5	M	No-affected	None	None	60	4.3	0.9	0.9	97	No	No

*NYHA* New York Heart Association Classification, *LVEF* left ventricular ejection fraction, *LVEDD* left ventricle end-diastolic dimension; *LVESD* left ventricle end-systolic dimension, *IVSD* interventricular septum thickness at end-diastole, *pro-BNP* B-type natriuretic peptide, *HTN* hypertension.

### Whole-exome sequencing

Genetic evaluation through the WES technique was performed for the proband (identified with an arrow in Fig. 1). NGS was performed using an MGI-seq platform to sequence approximately 70 million reads and with a quality score ≥ 20. Totally, more than 95% of the targeted regions were sequenced with a sensitivity of > 99%, using the platform. Additionally, the detection of targeted regions with flanked sequences is possible through the approach. In order to filter the variants, the raw output was filtered based on the frequency and functional effects of the variants. Variants that are likely to affect protein function (non-synonymous, stop gain, stop loss, frameshift deletions and insertions, and splice site variants) were labelled as functional variants. For further analysis, in order to remove common variants presented in reference genomes and databases, variants with a minor allele frequency lower than 1% were selected (Supplementary Table 1). Moreover, the effect of nonsynonymous missense variants was predicted using VarSome. After variant filtering, the assessment of variants was carried out by computational prediction software and much larger population databases such as genomAD. Moreover, filters were applied against published databases, and novel variants suspected to be destructive for proteins were chosen as candidates for further investigations. The evidence suggests that the variants may causes changes in the tissue expression of proteins. Finally, international valid databases including MalaCards, OMIM, and GeneCards were used regarding the confirmation process. Furthermore, predicting software including Mutation Taster, SIFT, DANN, and FATHMM were used so as to foresee how the missense variant may affect the biological function of protein. Finally, the candidate gene was selected based on the applied filters and comparison against the cardiomyopathy gene list (Supplementary Table 2).

### Variant validation studies using Sanger sequencing

According to WES annotation, a variant with uncertain significance (VUS) in exon 2 of the *GATA6* gene was found. Then, the variant was confirmed in the index patient, using the PCR method in addition to the Sanger sequencing technique. The sequences of PCR primers, for a product size of 363 bp, were as follows:

Forward primer: 5′-GCGCTTCCCCTACTCTCC-3′ and reverse primer: 5′-CGACCCTTACCTGCACTGG-3′. Furthermore, segregation studies were performed for both non-affected and affected people in the pedigree. We performed PCR under the following conditions:

Mastermix 2× containing of a solution of DNA polymerase, deoxyribonucleotide triphosphates, reaction buffer, and *Taq* DNA polymerase was used in addition to, MgCl2, and 10 pM of forward and reverse primers, 100 ng DNA; the volume in which the reactions were done was 25 μl. The initial cycle of denaturation was performed for 5 min at 95 °C, in addition to 35 more cycles at 95 °C for 30 s, annealing at 60 °C for 30 s, and extension for 30 s at 72 °C, plus a final 7 min extension at 72 °C. In the end, electrophoresis through agarose gel was used to check the products of PCR (Supplementary Fig. 1), and they were sent for sequencing using the Sanger technique. Snap gene software was used to analyse the gene sequences.

### Tertiary structure prediction of the *GATA6* and verification

We built the protein-model of both the wild-type of *GATA6* and mutant, aiming to assess the impact of the H329Y mutant on the structure of the wild-type of *GATA*6 with I-TASSER.

Using a threading modelling methodology on the I-TASSER online prediction web server (https://zhanglab.ccmb.med.umich.edu/I-TASSER/), we predicted the modelling of the 3D structures of the wild-type of the *GATA6* and H329Y mutant. We used I-TASSER to determine the 3D structure of the protein. In this case, primary amino acid sequences of the selected wild-type of the *GATA6* and H329Y mutant were considered, as well as using the iterative simulations of segmentation assembly. To determine threading-alignment quality, predicted alignment of secondary structure components for target sequences and template were analysed. The I-TASSER server is believed to be the best-ranked method in order to predict the structure of protein. The method includes a critical evaluation of Protein-Structure Prediction^31–37^. I-TASSER, Z score and normalized Z-score > 1, depicts a template associated by a good-alignment quality. The convergence and threading alignment of structure refinement simulations is reflected by the Confidence-score (values − 5 to 2) with higher score which corresponds to a high-confidence model. The accurate topology of the model is indicated by the template modelling score (> 0.5); on the other hand, score (< 0.17) is responsible for a model of random topology^32^. The best-ranked model is chosen based on the described scores. As a result, we chose models having the highest Z-score, Confidence-score and TM-score for further assessments. We used the SAVES server (https://saves.mbi.ucla.edu/) so that we could evaluate the quality of the structural models which were generated by I-TASSER. Numerous tools, including ERRAT, and Ramachandran plot analysis are used regarding the evaluation of the quality of the model. Investigating the PSi/Phi Ramachandran plot analysis using the saves server invest, the accuracy of the predicted models was evaluated^38^. ERRAT is a well-known program, by which the protein structures are verified based on atomic interactions^39^. Considering the root mean square deviation (RMSD) and TM-score values, the best and the most meticulous structure was selected^40^. 3D structures of the wild-type *GATA6* and mutant proteins were visualized, using PyMOL^41^.

### Molecular dynamics simulations

We used the GROMACS package version 5.0.7 (GROMOS96 43a1) in order to perform the molecular dynamics simulation of the wild-type structure and the H329Yvariant, which was expected to provide us the thorough comparison of structural changes within time^42^. The 3D structures were placed in the central zone of a cubic cell, surrounded by simple point charge extended (SPC/E) water with a box edge set at 1.0 nm (nanometre)^43^. In the next step, 7 chloride ions were added, aiming to neutralize the overall charge of the system. The position of protein and heavy atoms was controlled. Moreover, the protein and heavy atoms and simulations were recruited for NVT equilibration ensemble. We used velocity rescaling method to keep the number of particles, the volume of the system and the temperature t at 310.15 K for 100 ps (Pico seconds) constantly^44^, followed by 100 ps of NPT equilibration ensemble, in which the number of particles, the pressure and the temperature were kept constant at 1 bar. Nose–Hoover thermostat was used to control the temperature, and the pressure was controlled, using Parrinello-Rahman barostat. After doing well-equilibration of the system, we ran a 50 ns (nanoseconds) of MD simulation for the wild-type *GATA6* and the H329Y mutant structures. We used time-step of 2 fs (femtosecond), where all bonds were constrained. The process was performed by the linear constraint solver (LINCS) algorithm^45^. As described previously, we used the analysis of Root-mean-square deviation (RMSD), Radius of gyration (Rg), Root-mean-square fluctuation (RMSF), and Number of hydrogen bonds (HB) to evaluate conformational changes and stability in the wild-type *GATA6* protein and the H329Y mutant^46^. The EXCELL tool was used to plot all graphs.

## Results

Clinically, there were four affected individuals in the family, all of whom had early-onset symptoms of FDCM, including left ventricular enlargement in addition to severe systolic dysfunction. Based on the 2016 ESC/EAS guidelines, they fulfilled the criteria of FDCM.

First, the results of the WES test in the index patient were filtered. We excluded the patient`s common single nucleotide polymorphisms (SNPs), which were not pathogenic based on databases, in addition to variants whose frequency was more than 1%. A rare protein-altering variant in the *GATA6* gene (c.985C > T (NM_005257.6)) in exon 2 placed on chromosome 18 was identified in the index (III4) patient. Based on the ACMG/AMP 2015 guidelines, this variant was reported to be VUS and it is clinically interpreted as PM2, PP3 (Supplementary Table 3). The variant was present in the all affected individuals in the pedigree; on the other hand, it was not detected in any of the unaffected ones. The data resulted from the genotyping of His329Tyr variant in 50 healthy individuals, revealed that the mutation was absent in the control group.

**Figure 2:**
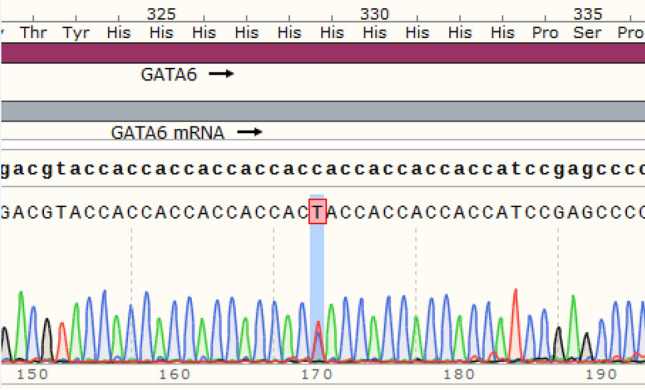
DNA sequence of a segment flanking H329Y in *GATA6* gene from an affected member. Histidine converts to Tyrosine following a single base substitution; the substitution of a Histidine by Tyrosine takes place at position 329. Red highlight denotes a heterozygote C > T nucleotide substitution.

Moreover, the frequency of this variant was investigated in several databases, such as ExAC, genomAD, 1000 Genomes, ESP, and Iranome; nevertheless, we failed to indicate the definite frequency. This mutation is a substitution of a Histidine by Tyrosine at position 329 of the *GATA6* gene (p.H329Y) (Fig. 2). Furthermore, this variant is conserved among paralogues and orthologues in some species such as Ptroglodytes, Mmulatta, Mmusculus, Dmelanogaster and humans. This variant is known as disease-causing variant in prediction software such as SIFT, Mutation taster, FATHMM and DANN (Supplementary Table 4).

### Prediction of 3D structures and validation

**Figure 3:**
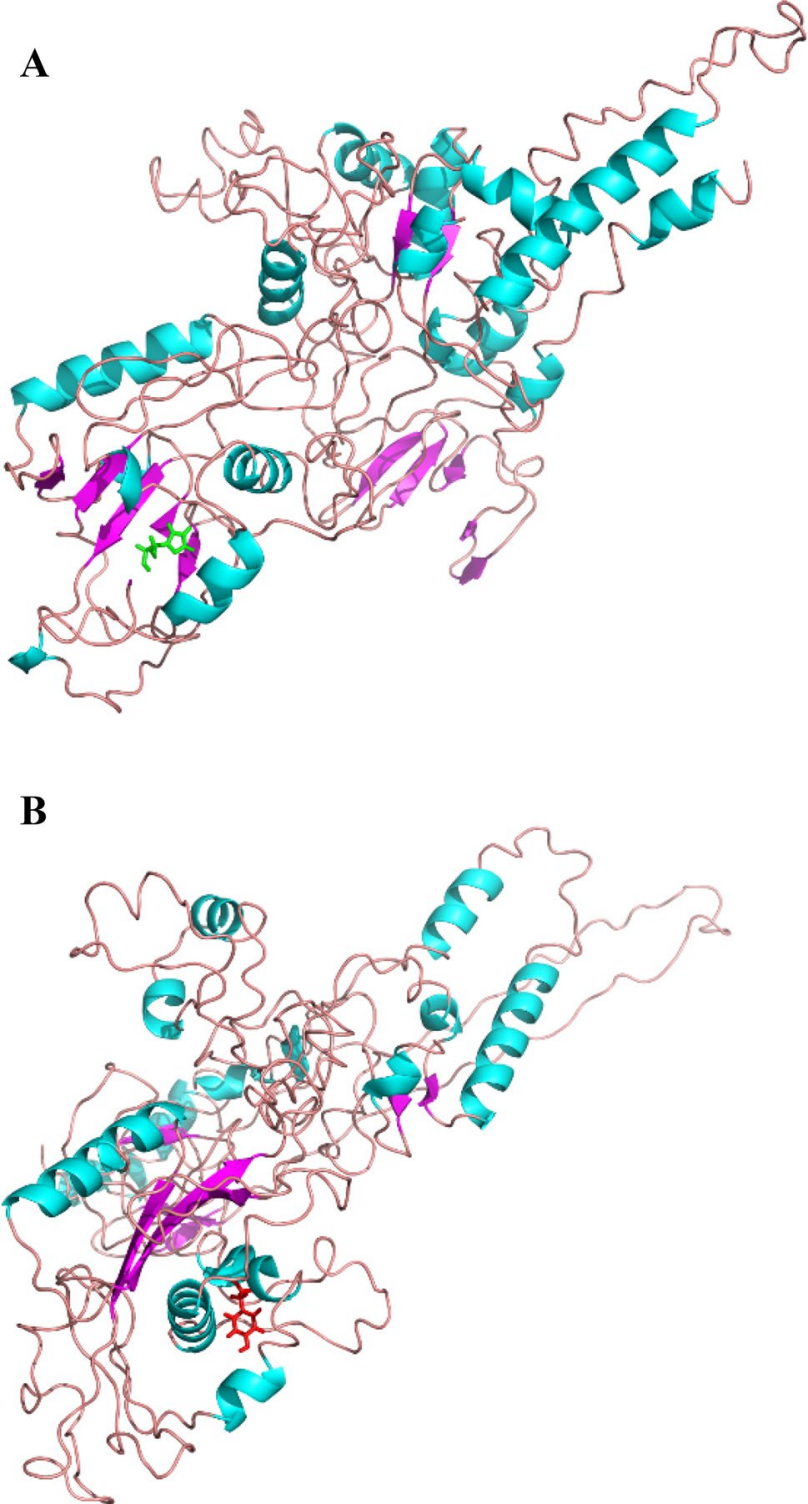
(**A**) In silico modelled structure of the wild-type of *GATA6*. The 3D model of the wild-type of *GATA6* generated using I-TASSER. The residue His329 is highlighted in stick model (Green colour). The α‐helices are represented by cyan ribbons, the β‐strands are represented by magentas arrows, and the coiled regions are represented in light pink. (**B**) The residue Tyr329 in the H329Y variant is highlighted in stick model (red colour).

I-TASSER identified human Crystal structure of the full DNA binding domain of *GATA3*-complex 2 as the most similar structural templates (pdb ID: 4hc7) for modelling of the wild-type of *GATA6* and the H329Y mutant. Top ranking models with highest C-scores: − 0.97 and − 1.50, respectively for the wild-type of *GATA6* and the H329Y mutant models are shown in Fig. 3A,B. The TM-score of the wild-type of *GATA6* and the H329Y mutant models was 0.59 ± 0.14 and 0.53 ± 0.15 respectively. Ramachandran plots were obtained from PROCHECK, which is algorithms that check the overall stereochemical quality of a protein structure. The plots show the phi(F)-psi(ψ) torsion angles for every residue of a protein. As PROCHECK’s Ramachandran plot indicates, the most favoured regions consist of 55.3% and 56.4% of residues for the wild-type of *GATA6* and mutant, respectively; 33.8% of them are lied in additional allowed regions and 4.4% in generously allowed regions (regarding the wild-type). 32.4% of them are also lied in additional allowed regions and 7.8% in generously allowed regions (regarding the mutant). Finally, 6.4% and 3.3% are placed in regions which are disallowed for the wild-type of *GATA6,* and H329Y mutant model, respectively*.* The ERRAT plot for the models of the wild-type of *GATA6* and the H329Y mutant exhibited overall quality factors of 77.12% and 68.19%, respectively, further supporting its quality as scores higher than 50 are considered acceptable. Furthermore, the results of validation of structural models were further verified through overall quality factor of ERRAT score and Ramachandran plot which indicated good quality of the models.

### Molecular dynamics simulation studies of the *GATA6*

All-important statistical results of the simulations were summarized in the Table 2.

**Table 2:** Analysis of MD trajectory of the wild-type of *GATA6* and the H329Y mutant.

Type of variant	RMSD		Radius of gyration		Intra molecular H-bonds		RMSF	
	Range	Average	Range	Average	Range	Average	Range	Average
Wild-type	0–1.262	1.008	6.901–6.97	6.937	247–373	330	0.122–1.068	0.33
H329Y mutant	0–1.453	1.218	2.476–3.114	2.682	216–386	331	0.135–1.147	0.40

**Figure 4:**
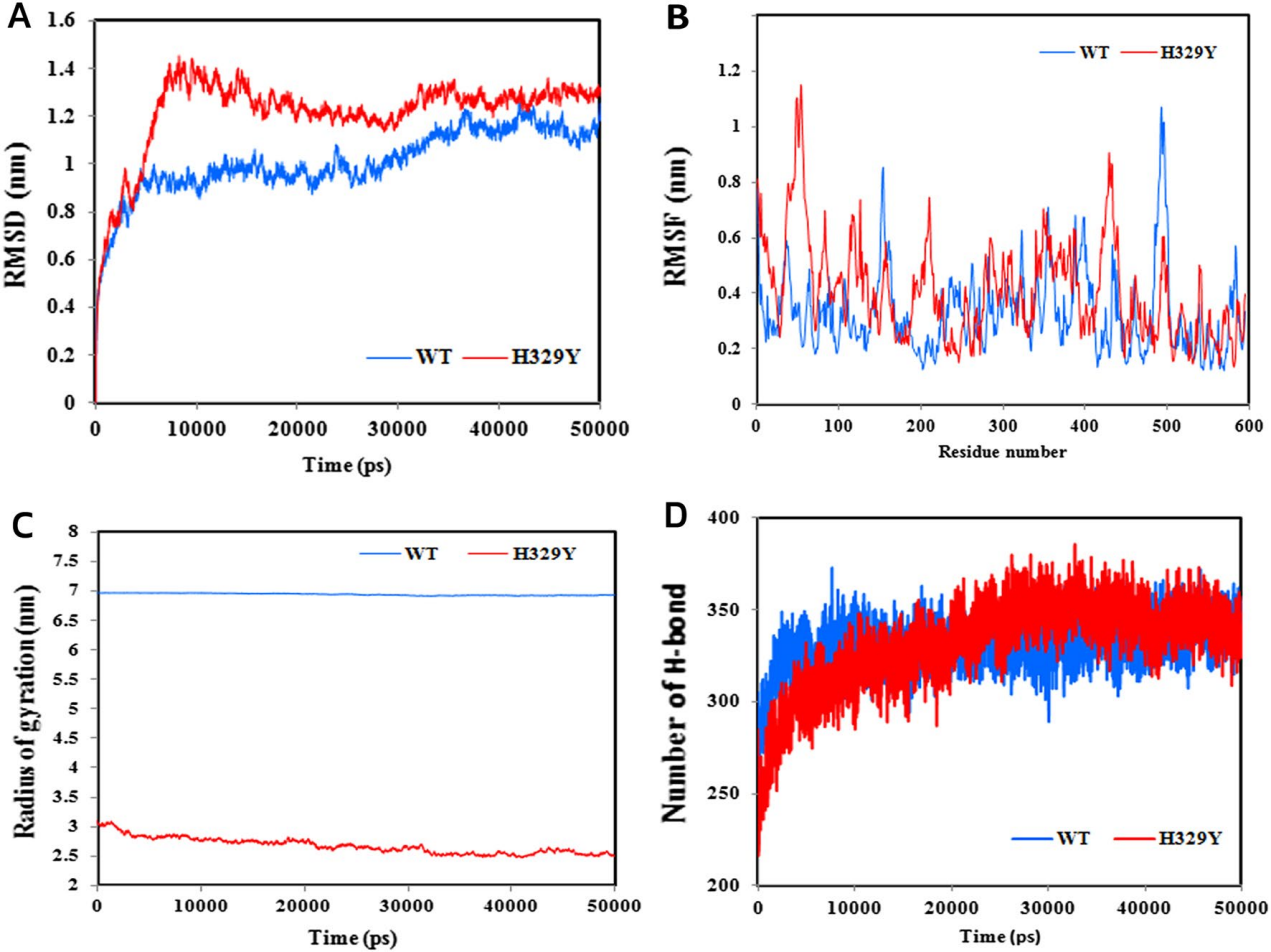
(**A**) Backbone root-mean-square-deviation (RMSD) of the wild-type of *GATA6* and the H329Y mutant protein for 50,000 ps molecular dynamics simulation. The wild-type of *GATA6* is represented in blue, and mutant H329Y in red. (**B**) RMSF diagram of *GATA6* protein backbone atoms and mutant H329Y. The root-mean-square fluctuation for each residue of *GATA6* is shown. (**C**) Radius of gyration of *GATA6* protein and the H329Y mutant. The Radius of gyration of Cα atoms of the wild-type of *GATA6* and the H329Y mutant during the MD trajectory is shown. (**D**) Total number of intramolecular hydrogen bonds for *GATA6* protein and the H329Y mutant.

In order to understand the structural changes and the effects of the H329Y mutation on the wild-type protein structure of *GATA6*, the RMSD for the backbones of the wild-type of *GATA6* and the H329Y mutant was calculated during the simulations (Fig. 4A).

We calculated the root mean square deviation, aiming to predict the protein-stability. The calculated RMSD value from the Cα atoms from the initial structure of protein, is shown in Fig. 4A. The deviation shows the level of stability in the protein-structure within a time scale simulation. The higher RMSD is, the higher stability will be, which can result in achieving higher protein rigidity and vice versa. As represented by Fig. 4A, the whole pattern of the RMSD is changed owing to the mutation. The average RMSD value for the wild-type of *GATA6* and the H329Y mutant is 1 and 1.2 nm respectively. The RMSD value for wild-type of *GATA6* and the H329Y mutant was increased till 36,000 ps but after that time period the RMSD value was stable and showed a constant peak. The wild-type of *GATA6* with an RMSD of about 1 nm was stabilized, while RMSD values of the H329Y mutant were higher than wild-type it was stabilized with an RMSD of about 1.3 nm. The result suggested that the H329Y mutant is unstable compared to the wild-type of *GATA6*.

In order to determine the effects of mutation on the dynamic behaviour of protein residues, the RMSF (the root mean square average distance between an atom or a group of atoms and its average position in a given structure) fluctuations of each residue were investigated. Figure 4B shows that the residual surface fluctuations for the H329Y mutant were quite high compared to the wild-type of *GATA6*. The average RMSF value for the wild-type of *GATA6* and the H329Y mutant are 0.33 nm and 0.4 nm. It showed that the mutation inducing the structural effects in the residues region. The highest peak of the RMSF value greater than 0.8 nm were for residues at positions 1, 46–57 and 430. There was a significant difference in RMSF value located on residue 329. The wild type of *GATA6* showed a fluctuation of 0.25 nm at H329, whereas the fluctuation at the same position on mutant H329Y was 0.3 nm, which demonstrates the flexibility has been increased. Mutant H329Y causes a considerable increase in the RMSF value on the residue number 54 of the protein. Residue number 54 showed fluctuation values 0.3 nm in the wild-type, while in the mutant, this residue fluctuation values was 0.9 nm. Residues 42–56 showed fluctuation values ranging from 0.2 to 0.3 nm in the wild-type, while in the H329Y mutant, these residues fluctuation values ranged from 0.7 to 1.1 nm. So in the N-terminal domain the H329Y mutant showed higher fluctuation compare to the wild-type of *GATA6*. The result of fluctuation analysis suggested the greatest degree of flexibility by the H329Y.

According to Fig. 4C, the radius analysis of the wild-type of *GATA6* was performed with related mutation and compared with each other. When reviewing the statistical data from Table 2, We got an interesting result that the wild-type of *GATA6* had the highest gyration radius of 6.93 nm and the lowest structural compaction while the H329Y mutant had the lowest gyration radius of 2.68 nm and the highest structural compaction. These results indicate that the H329Y mutant protein undergoes fundamental structural changes compared to the wild-type protein. The Rg analysis showed that the wild type of *GATA6* simulation had a higher average Rg value (6.937 nm) than that in the H329Y mutant simulation (2.682 nm). This indicates that the wild-type of *GATA6* structure was less compact than H329Y variant. The H329Y mutant showed a constant Rg value of 2.682 nm throughout the simulation, which indicates a greater level of compaction when compared to the wild-type of *GATA6* structure. According to the Rg result, there is strong relationship between the RMSD result and the Rg result. The relationship suggests the increased compactness of the H329Y mutant owing to the mutation. Additionally, different conformation is associated with the mutant form.

To evaluate the stability of the wild-type of *GATA6* and reported mutation, the total number of intramolecular hydrogen bonds was analysed according to Fig. 4D. The hydrogen bonds appear to play a key role to maintain the protein-stability, stabilizing the protein in a definite manner. The more the number of hydrogen bonds is, the more compact structure will appear and vice versa. The average number of hydrogen bonds for the wild-type of *GATA6* and mutant are 330 and 331, respectively. Hydrogen bonds analysis in the wild-type of *GATA6* and H329Y mutant is essential to understand the stability and flexibility of the protein. Considerable variety exists regarding the intermolecular hydrogen frequency of the H329Y mutant structure and the wild-type of *GATA6* structure. Hydrogen frequency of the wild-type of *GATA6* structure on an average was ranging from 247 to 373. Whereas, the number of H329Y mutant hydrogen bonds ranged from 216 to 386. The moderately increasing number of the hydrogen bonds after 20,000 ps at the H329Y mutant was demonstrated by the plot. According to the increased number of hydrogen bonds following the mutation, it is predicted that we will have more compactness and rigidity in the mutant forms. Thus, the result seems similar to the Rg result.

## Discussion

Dilated cardiomyopathy is a complex cardiovascular disorder, which is known as a common leading cause of heart transplantation^47^. The aetiologies resulting in DCM are various, about 50% of them are considered idiopathic. Surprisingly, the most idiopathic causes are related to genetic factors, and research shows that even those individuals, affected by non-idiopathic causes, seem to be associated the influence of a genetic background^47–49^.

The recent advances in genetic diagnostic methods with interesting potentialities, varied from single base sequencing to whole-genome sequencing, have resulted in identifying a growing number of mutated genes and variants associating with familial disorders such as FDCM. Furthermore, cardiomyopathies have such a diversity that more suspicious genes and variants should be investigated so that we can provide a thorough and convincing genetic consultation for the patient, their family members, and their next generation. Considering the cost-effectiveness, wide coverage, and high sensitivity of WES, it is one of the most popular and powerful diagnostic tools in the field of molecular assessment^2,47^. Therefore, we chose the combination of cardiomyopathy-related gene-filtering and WES to investigate the potential genetic factors and mutations related to DCM in this Iranian family.

The results of our study confirmed the presence of a variant in the *GATA6* gene being suspicious of having a role in both formation and progression of early-onset FDCM amongst the affected patients in the pedigree. *GATA6*, encoding an essential zinc-finger transcription factor of cardiogenesis, has been well-known for its expression during the development of the heart in foetus^14,20,21,50–53^. LEI XU et al. identified two novel heterozygous mutations in *GATA6*, p.H475R, and p.C447Y, during their genetic evaluation on two families with DCM. The disease was inherited through an autosomal-dominant pattern amongst the family members, and it has a complete penetrance. Their functional assays suggested the association of the mutant *GATA6* proteins whose transcriptional activity was reduced significantly. As a result, they claimed that there might be a relationship between functionally compromised *GATA6* and DCM in the families^52^. However, more evidence is needed to prove the role of this gene in DCM.

It is established through research that genetic defects in the *GATA6* gene are capable of leading to DCM formation even in animals. For example, the depletion of *GATA6* in the embryo of zebrafish may cause various morphogenetic changes in the heart including partially fused tube, and fused but non-looping tube, and cardia bifida^54^. On the other hand, the depletion of *GATA4* and *GATA6* in the zebrafish embryos does not cause any damage, and restoring either gene product was sufficient to rescue cardiomyocyte specification^55^.

As Liang Q et al. claimed through their study on mice, cardiac hypertrophy may be present as a result of the overexpression of *GATA4* or *GATA6*^56,57^, while the cardiomyocyte-specific conditional deletion of *GATA6* can cause a significantly reduced hypertrophic response to the stimulation of pressure overload, rapidly leading to heart failure. The event was similarly observed in the mice having the heart-specific deletion of *GATA4*. Furthermore, the combinatorial deletion of *GATA4* and *GATA6* from the adult heart resulted in DCM and lethality by 16 weeks of age^57–59^. The evidence shows that *GATA6* plays a critical role in cardiac remodelling, and development. *GATA6* is not only involved in the regulation of cardiac gene expression alone, but also it can cooperate with its transcriptionally synergistic partners, including *NKX2-5, TBX20*, and *GATA4*. Indeed, the presence of functionally compromised *GATA6* is followed by DCM manifestations as the result of the poor expression of genes that are involved in cardiac development^21,60–65^.

In addition to the complex laboratory task of identifying disease associated variants, the effects of variants on the structure and function of proteins can be easily obtained using current in silico approaches. Studying the features of the structure of variant protein at the molecular level, the ability of making predictions on the final functional and structural effects caused by mutant variants will be earned^66^. Based on the criteria for evaluating the quality of the model in I-TASSER, PROCHECK’s Ramachandran plot and the quality factor in ERRAT, excellent 3D models for natural and mutant proteins were presented. The findings of molecular dynamics simulations are capable of providing accurate and detailed knowledge about particle motions as a function of time^67^.

Compared to wild-type in RMSF, RMSD, radius of gyration, and number of hydrogen bond, there was an obvious loss of stability in *GATA6* H329Y mutant. Molecular stability and flexibility changes from RMSF and RMSD were demonstrated through simulations. Stability is considered a basic and fundamental characteristic which plays a key role in the enhancement of biomolecular function, regulation, and activity. Moreover, it was understood that Structural mutations had some effects on buried residues located in the protein core, and the impact had caused changes in the size and charge of amino acid and hydrogen bond numbers^68^. The RMSD calculation is a global measurement, by which the two different conformations of a single protein are compared. Indeed, the calculation suggests detailed information demonstrating the differences between a protein’s backbones and its initial structural conformation into its last position^69–71^.

As shown in Fig. 4, the average RMSD value of H329Y is very close to the wild-type value but the RMSD simulation showed that the wild-type of *GATA6* maintained an overall stability throughout 50 ns of simulation while the H329Y mutant displayed more fluctuations. Molecular dynamics simulations of variants confirmed a good inverse correlation between protein stability and local flexibility which was determined by the magnitude of fluctuations with respect to the average conformations^72^. It is expected that not until the protein has a suitable structure can it provide a convincing function. According to the results earned by RMSD calculation, the stability of H329Y mutant is decreased, and protein geometry is altered as well. As far as the native function of protein is concerned, the proteins appear to lose their expected function following the mutation. As similarly confirmed by the previous studies, we can widely use molecular dynamics simulation, thereby appraising the impact of the mutations on specific amino acid residues. Apparently, defects in the flexibility and stability of the protein-structure is expected to cause the appearance of pathological phenotypes^73^. Not only will the rigorous phenotype prediction and thorough structural analysis of missense mutations through molecular dynamics simulation and in silico screening creatively develop the screening strategies for destructive and pathological mutations, but also provide new prospects for the rational design during recombinant productions as well as and tailored medicine. As shown in this study, occurrence of mutation and change in an amino acid has led to the creation of a new structure of *GATA6* protein (Fig. 3A,B), which is based on data obtained from various structural analyses of RMSD, RMSF, radius of gyration, and number of hydrogen bond (Fig. 4A–D). According to some reports^74,75^, these changes could have an implication on the phenotype which was assayed in this study via in silico simulation. This study offers a detailed insight to elucidate the pathogenic effects of nonsynonymous single nucleotide polymorphisms (nsSNPs) of *GATA6* and the possible consequences of this variation. MD simulation analyses, especially radius of gyration indicated a significant conformational loss in *GATA6* protein structure due to H329Y mutation. These results provide further support for the hypothesis that any significant changes, especially an increase in the RMSD and RMSF value, high fluctuations, an increase or decrease in flexibility, loss or gain of intracellular forces of noncovalent, or significant changes in protein compression levels during molecular dynamics simulations can be used as criteria to evaluate the types of mutations. This would help to filter and classify novel variants as low-risk or deleterious high-risk variants in other proteins. According to these data, we can infer that computational prediction of the potential effects of mutations, will be providing a fast and cost-effective strategy compared to complex laboratory evaluations for classifying novel variants in the future. For instance, the potential effect of Riluzole and Edaravone, two FDA approved drugs for amyotrophic lateral sclerosis (ALS), that are known to play a key role in regards to the stabilization and structural deviations of the mutant profilin-1 gene (*PFN-1*) is assessed by in silico tools. Considering these analyses, the structural changes of mutant PFN-1 protein are revealed. The changes are likely to suggest a convincing explanation regarding the neurotoxicity and the reason(s) for possible loss and gain of function of PFN-1 in the neurotoxic model of ALS^76^.

The RMSF analysis is considered a beneficial tool representing local flexibility differences among residues, using the molecular dynamics simulation^67^. The higher RMSF values are, the more flexible movements are expected; in contrast, lower RMSF values are followed by more limited movements during simulation related to average positions^77^. According to Fig. 4B, The RMSF result suggested that mutation alters the structural flexibility based on achievement of the higher degree of RMSF compared to the wild type of *GATA6.* The evidence shows that flexibility alteration which results from the impact of H329Y mutant, particularly involves the N-terminal end, and the tendency of flexibility loss may interrupt the function of native protein. This also accords with recent studies indicating that communication between stability, flexibility, and activity is complex. In other words, if the protein can run its original function in addition to maintaining its structural integrity, a combination of structure rigidity and a certain degree of flexibility seems to be required^78^. Accordingly, functional assessment of the single nucleotide polymorphisms with Solute Carrier Family 26 Member 4 (*SLC26A4*) gene was performed in the another research, which led to confirm the ability of the molecular dynamics simulation methods^27^.

The radius of gravity of a protein is a measure of its density and compaction, and shows the extent to which the total distance of the protein atoms from the centre of mass of the protein fluctuates. Radius analysis provides comprehensive information for studying the overall dimensions of the structure^67,79^. During protein folding, the amount of germination remains constant, but during the protein unfolding process, its amount changes over time. The radius of gyration indicates the compactness of protein structure, where higher values denote less compactness and lower values denote higher compactness^80^. The H329Y mutant shows a constant Rg value of 2.682 nm throughout the simulation, presenting a greater compaction level of the wild-type, which the Rg value was 6.937 nm. The radius of gyration analysis is also in well agreement with the RMSF result. Some proteins lose their expected function due to the missense mutations in the genes^73,81^.

The damaging, destabilizing, and deleterious effect of the mutation R142Q on Tau‐tubulin kinase1 (*TTBK1*) structure was comprehensively highlighted by a recent research study through molecular dynamics simulations and computational predictions. These findings demonstrate that the presence of R142Q mutation on *TTBK1* is accountable for the instability of the structure, and it may disrupt its biological functions. It is expected that the mutation will be used as a diagnostic marker regarding the treatment of Alzheimer`s disease in the future^82^. The crucial role of several noncovalent interactions, such as hydrogen bonding, van der Waals hydrophobic, and electrostatic interactions in maintaining stability to protein structures is well-established. In order to understand the changes on protein structure, which are caused by deleterious mutations, evaluating the alterations in hydrogen bond pattern appears essential^83^. The findings of hydrogen bond assessment suggested significant variation in intermolecular hydrogen frequency of the H329Y mutant structure in comparison with the wild-type of *GATA6* structure. As shown in Table 2 and Fig. 4D the wild-type of *GATA6* and the H329Y mutant structures displayed hydrogen frequency on an average ranging from 247 to 373 and ranging from 216 to 386, respectively. According to a recent study, the effects of all nsSNPs on the protein structure were evaluated through the comparison of the hydrogen and hydrophobic interactions in the wild type Stxbp1 gene structure and its mutant forms. These findings demonstrate that the all nsSNPs affect the protein structure on different levels^84^.

These results are in agreement with those obtained by studying the prediction of protein structure and molecular dynamics simulation which were done to confirm the impact of mutation on the protein structure and function^27^. The evidence earned by the analysis of RMSD, RMSF, and Rg, and hydrogen bond after a molecular dynamics simulation demonstrated that pathogenic Single Nucleotide Polymorphisms may influence the flexibility, stability, and all characteristics of proteins, as though the whole structure of proteins is affected. As a result, the original function of the protein will be disrupted^81^. According to the results obtained from in total 50 ns MD simulations, it can be concluded that the H329Y mutation in the *GATA6* gene may induce phenotypic damages, thereby being associated disease formation. There were great commonalities between our simulations and simulations performed through the previous experiments. Thus, our MD results are strongly considered accurate and reliable.

There can be no doubt that the critical role of the *GATA* family in cardiac tissue cannot be ignored, whether in the case of congenital heart diseases or cardiomyopathies. Our growing knowledge about the genetic basis of DCM owing to the recent advances of bimolecular genetic diagnostic tests, including WES has provided valuable insight for us so that we can set our goals to investigate further information through later studies. Having improved understanding of the genetic background of cardiac disease, we will be capable of predicting the disease recurrence in the next generation, thereby providing a proper genetic consultation for our patients.

## Conclusions

Several variations have been identified in the *GATA6* gene through the previous studies. Our findings suggested a novel missense mutation in the exon 2 of the *GATA6* gene (p. H329Y) as well. The functional and structural consequences of the mutation have not been analysed yet to emphasize their role regarding DCM. Yet, the definite mechanism of disease formation should be further investigated. It is recommended that molecular sequencing appraisal through large cohort studies be performed to provide a thorough body of information about the genetic basis of FDCM. In brief, this variant should be considered as an important candidate, causing DCM.

Computational methods and in silico modelling, along with laboratory studies, appear to be beneficial in predicting the effects of harmful point mutations on protein structure and function, as well as the effects of various factors on flexibility, stability, and protein compaction. The flexibility loss is observed in RMSF plot in the H329Y mutant structure. The MD simulation results, considering RMSD plot, suggest the decrease of protein-stability following the mutation, compared to the wild-type protein. The results of the Rg plot analysis demonstrated the increase in compactness of the H329Y mutant structure. As the evidence suggests, the original geometry of the structure is changing in the wild-type protein following the mutation. In addition to producing a major effect on the structural conformation of *GATA6* protein, the function of *GATA6* protein will be affected, subsequently. In fact, an appropriate conformational geometry is understood to be important in order that the protein can perform its native function. However, our results show that the mutation results in an unstable and compact structure of the protein.

## Supplementary Information

Supplementary Information.

## Abbreviations

FDCM: Familial dilated cardiomyopathy
NGS: Next generation sequencing
WES: Whole-exome sequencing
SCD: Sudden cardiac death
LVEF: Left ventricular ejection fraction
LVEDD: Left ventricle end-diastolic dimension
LVESD: Left ventricle end-systolic dimension
NYHA: New York Heart Association Classification
IVSD: Interventricular septum thickness at end-diastole
CHDs: Coronary artery diseases
BNP: B-type natriuretic peptide
bp: Base pair
Rg: Radius of gyration
RMSD: Root-mean-square deviation
RMSF: Root-mean-square fluctuation
ns: Nanoseconds
ps: Picosecond
HB: Number of hydrogen bonds
MD: Molecular dynamics
3D: Three-dimensional
LINCS: Linear constraint solver
nm: Nanometre
nsSNPs: Nonsynonymous single nucleotide polymorphisms
ALS: Amyotrophic lateral sclerosis
