# Supplementary material for: A novel causative functional mutation in GATA6 gene is responsible for familial dilated cardiomyopathy as supported by in silico functional analysis
Source: Sci Rep. 2022 Aug 12;12:13752. doi: 10.1038/s41598-022-13993-6 (PMC9374661; doi:10.1038/s41598-022-13993-6)
Supplement: Supplementary file 1 — Supplementary Information. [file 41598_2022_13993_MOESM1_ESM.pdf]

A novel causative functional mutation in *GATA6* gene is responsible for familial dilated cardiomyopathy as supported by *in silico* functional analysis

Afrouz Khazamipour[1], Nazanin Gholampour-Faroji[2], Tina Zeraati[3], Farveh Vakilian[4], Aliakbar Haddad-Mashadrizeh[5], Majid Ghayour Mobarhan[6*], Alireza Pasdar[1,3,7,8*]

1) Department of Medical Genetics and Molecular Medicine, Faculty of Medicine, Mashhad University of Medical Sciences, Mashhad, Iran
2) Biotechnology Department, Iranian Research Organization for Science and Technology (IROST), Tehran, Iran
3) Medical Genetics Research Centre, Faculty of Medicine, Mashhad University of Medical Sciences, Mashhad, Iran
4) Department of Cardiology, Preventive Atherosclerotic Research Center, Imam Reza Hospital, Faculty of Medicine, Mashhad University of Medical Sciences, Mashhad, Iran
5) Industrial Biotechnology Research Group, Institute of Biotechnology, Ferdowsi University of Mashhad, Mashhad, Iran
6) Metabolic Syndrome Research Centre, School of Medicine, Mashhad University of Medical Sciences, Mashhad, Iran
7) Division of Applied Medicine, Medical School, University of Aberdeen, Foresterhill, Aberdeen, UK
8) Bioinformatics Research Group, Mashhad University of Medical Sciences, Mashhad, Iran

*Correspondence:

Majid Ghayour-Mobarhan, Metabolic Syndrome Research Centre, School of Medicine, Mashhad University of Medical Sciences, Mashhad, Iran.
Email: ghayourm@mums.ac.ir

Alireza Pasdar, Department of Medical Genetics, Faculty of Medicine, Mashhad University of Medical Sciences, Mashhad, Iran.
Email: PasdarA@mums.ac.ir

Supplementary Table 1: Variants filtering steps of the whole exome sequencing

DCM- III4	
Total variants	144082
Exonic & Splice variants	24483
Non-synonymous, insertion/deletion, frameshift and splice variants	11369
Variants with MAF* <0.01 in public databases	105
Heterozygous variants	52
Checked against DCM panel & other cardiomyopathy causing genes♯	1<br>(*GATA6*)

*MAF, minor allele frequency

♯ (Supplementary Table 2)

Supplementary Table 2: Cardiomyopathy-related gene list (296 gene)

AARS2	*ABCC6*	*ABCC9*	*ACAD9*	*ACADVL*	*ACTA1*	*ACTA2*	*ACTC1*	*ACTN2*	*ACVR2B*	*ACVRL1*
AGK	*AGL*	*AKAP9*	*ALMS1*	*ALPK3*	*ANK2*	*ANO5*	*ANKRD1*	*APOA1*	*ARHGAP31*	*ATM*
B3GAT3	*BAG3*	*BCOR*	*BMPR2*	*BRAF*	*CACNA1C*	*CACNB2*	*CALM1*	*CALM2*	*CALR3*	*CAPN3*
CASQ2	*CAV3*	*CAVIN4*	*CBL*	*CDH2*	*CFAP53*	*CFC1*	*CHD7*	*CITED2*	*CLDN16*	*CLDN19*
CNNM2	*COL1A1*	*COL1A2*	*COL3A1*	*COL4A1*	*COL4A2*	*COL5A1*	*COL5A2*	*COX15*	*CPT2*	*CREBBP*
CRELD1	*CRYAB*	*CRPPA*	*CSRP3*	*CTNNA3*	*DBH*	*DES*	*DMD*	*DNAJC19*	*DOLK*	*DSC2*
DSG2	*DSP*	*DTNA*	*DYSF*	*EEF1A2*	*EFEMP2*	*EGF*	*EHMT1*	*ELAC2*	*EMD*	*EPG5*
ETFA	*ETFB*	*ETFDH*	*ELN*	*ENG*	*EP300*	*EVC*	*EVC2*	*EYA4*	*FBN1*	*FBN2*
FBXO32	*FHL1*	*FKRP*	*FKTN*	*FLNA*	*FLNC*	*FOXC1*	*FOXD4*	*FOXF1*	*FOXH1*	*FOXRED1*
FXN	*FXYD2*	*GAA*	*GATA4*	*GATA5*	*GATA6*	*GATAD1*	*GBE1*	*GDF1*	*GDF2*	*GFM1*
GJA1	*GJA5*	*GLA*	*GLB1*	*GMPPB*	*GPC3*	*GPD1L*	*GTPBP3*	*GUSB*	*HADHA*	*HAND1*
HCCS	*HCN4*	*HFE*	*HRAS*	*HTRA1*	*ILK*	*JAG1*	*JPH2*	*JUP*	*KCNA1*	*KCNA5*
KCND3	*KCNE1*	*KCNE2*	*KCNE3*	*KCNH2*	*KCNJ2*	*KCNJ5*	*KCNK3*	*KCNQ1*	*KDM6A*	*KMT2D*
KRAS	*LAMA4*	*LAMP2*	*LARGE1*	*LDB3*	*LMNA*	*LRRC10*	*LZTR1*	*MAP2K1*	*MAP2K2*	*MED12*
MED13L	*MEIS2*	*MFAP5*	*MIB1*	*MLYCD*	*MMP21*	*MMP3*	*MTO1*	*MYBPC3*	*MYBPHL*	*MYH11*
MYH6	*MYH7*	*MYL2*	*MYL3*	*MYL4*	*MYLK*	*MYLK2*	*MYO6*	*MYOZ2*	*MYOT*	*MYPN*
NDUFAF2	*NEBL*	*NEXN*	*NF1*	*NIPBL*	*NKX2-5*	*NKX2-6*	*NODAL*	*NOTCH1*	*NOTCH2*	*NOTCH3*
NPPA	*NR2F2*	*NRAS*	*NSD1*	*PCCA*	*PCCB*	*PDLIM3*	*PKD1L1*	*PKD2*	*PKP2*	*PLEKHM2*
PLEC	*PLN*	*PNPLA2*	*PPA2*	*PPP1CB*	*PRDM16*	*PRKAG2*	*PRKG1*	*PSEN1*	*PSEN2*	*PTPN11*
RAF1	*RASA1*	*RASA2*	*RBCK1*	*RBM10*	*RBM20*	*RIT1*	*RMND1*	*RRAS*	*RYR2*	*SALL1*
SALL4	*SCN10A*	*SCN1B*	*SCN2B*	*SCN3B*	*SCN4B*	*SCN5A*	*SCNN1B*	*SCNN1G*	*SCO2*	*SDHA*
SELENON	*SEMA3A*	*SGCA*	*SGCB*	*SGCD*	*SGCG*	*SHOC2*	*SKI*	*SLC12A3*	*SLC22A5*	*SLC25A4*
SLC2A10	*SLC25A20*	*SLMAP*	*SMAD3*	*SMAD4*	*SMAD6*	*SMCHD1*	*SMC3*	*SNTA1*	*SOS1*	*SOS2*
SPEG	*SPRED1*	*SOX2*	*STRA6*	*SYNE1*	*SYNE2*	*TAB2*	*TAFAZZIN*	*TAZ*	*TBX1*	*TBX20*
TBX5	*TCAP*	*TFAP2B*	*TGFB2*	*TGFB3*	*TGFBR1*	*TGFBR2*	*TLL1*	*TMEM43*	*TMEM70*	*TNNC1*
TNNI3	*TNNI3K*	*TNNT2*	*TOR1AIP1*	*TPM1*	*TRDN*	*TREX1*	*TRIM32*	*TRIM63*	*TRPM4*	*TRPM6*
TSFM	*TTN*	*TTR*	*VCL*	*VCP*	*VPS13A*	*XK*	*ZEB2*	*ZFPM2*	*ZIC3*	

Considering the cardiomyopathy gene panels indicated in the following websites:

"https://www.ncbi.nlm.nih.gov/gtr/tests/552712/"

"https://www.centogene.com/science/centopedia/ngs-panel-genetic-testing-for-dilated-cardiomyopathy.html"

Supplementary Table 3. Variant Interpretation Criteria according to the ACMG/ClinGen Guidelines for the DCM Precision Medicine Study guidelines:

Gene/variant	Variant class	Strength	Specification type	Rule abbreviation	Rule description
GATA6 c.985C>T p.H329Y	VUS (Uncertain significance)	Moderate	DCM gene specification, study specific	PM2	Absent from gnomAD or at extremely low frequency in all gnomAD non-founder populations with available data
		Supporting	Method specification	PP3	Computational evidence support a deleterious effect (REVEL score >0.7)

Supplementary Table 4: Characteristics of H329Y mutation in *GATA6* gene based on the software analysis

No.	Prediction	Status
**1**	SIFT	Damaging
**2**	Mutation taster	Disease-causing
**3**	Provean	Neutral
**4**	FATHMM	Damaging
**5**	FATHMM-MKL	Damaging
**6**	MetalR	Damaging
**7**	MetaSVM	Damaging
**8**	DANN	Damaging

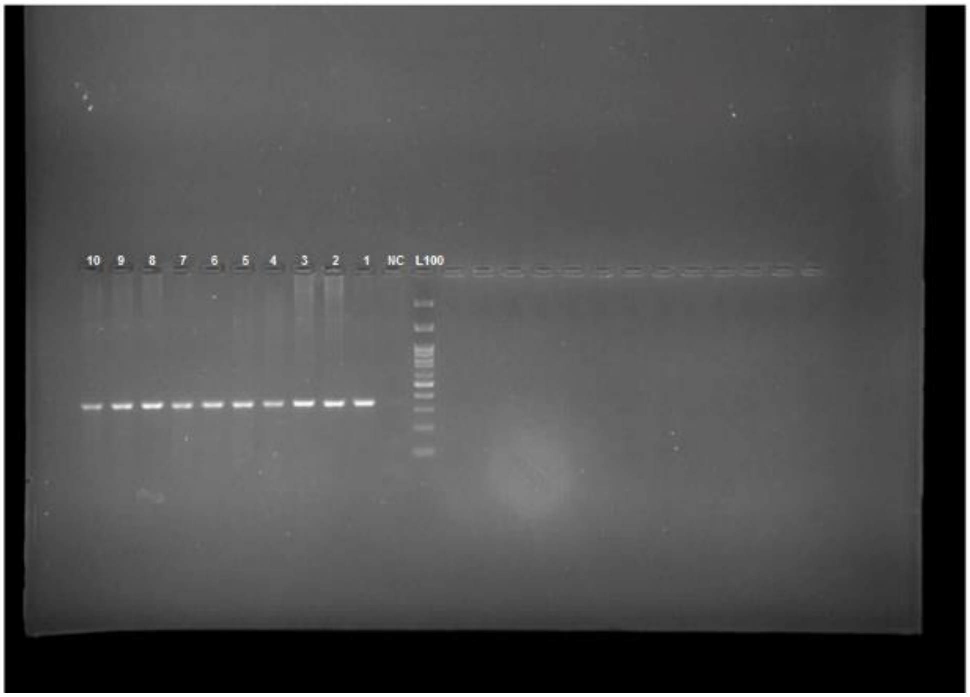

Supplementary Fig. 1: A PCR product with 363 bp length. L, Ladder; N.C, negative control; Lanes 1- 4 are the products of PCR for patient III-3, III-4, IV-2, II-4 and Lanes 5-8 are patient III-3, III-4, IV-2, II-4 (duplicate). Lanes 9 & 10 are for healthy family members. Other lanes on the right of the ladder are left blank.
